# Temperature-Dependent Self-Powered Solar-Blind Photodetector Based on Ag_2_O/β-Ga_2_O_3_ Heterojunction

**DOI:** 10.3390/nano12172983

**Published:** 2022-08-29

**Authors:** Taejun Park, Sangbin Park, Joon Hui Park, Ji Young Min, Yusup Jung, Sinsu Kyoung, Tai Young Kang, Kyunghwan Kim, You Seung Rim, Jeongsoo Hong

**Affiliations:** 1 Department of Electrical Engineering, College of IT Convergence, Gachon University, 1342, Seongnam-daero, Sujeong-gu, Seongnam-si 13120, Korea; 2 Department of Intelligent Mechatronics Engineering and Convergence Engineering for Intelligent Drone, Sejong University, 209, Neungdong-ro, Gwangjin-gu, Seoul 05006, Korea; 3 PowerCubeSemi, Inc., 686, Cheonggyesan-ro, Sujeong-gu, Seongnam-si 13105, Korea

**Keywords:** Ag_2_O/β-Ga_2_O_3_, heterojunction, deep ultraviolet, photodetector, post-annealing

## Abstract

In this study, a high-photoresponsivity self-powered deep ultraviolet (DUV) photodetector based on an Ag_2_O/β-Ga_2_O_3_ heterojunction was fabricated by depositing a p-type Ag_2_O thin film onto an n-type β-Ga_2_O_3_ layer. The device characteristics after post-annealing at temperatures ranging from 0 to 400 °C were investigated. Our DUV devices exhibited typical rectification characteristics. At a post-annealing temperature of 300 °C, the as-fabricated device had a low leakage current of 4.24 × 10^−11^ A, ideality factor of 2.08, and a barrier height of 1.12 eV. Moreover, a high photo-responsivity of 12.87 mA/W was obtained at a 100 μW/cm^2^ light intensity at a 254 nm wavelength at zero bias voltage, the detectivity was 2.70 × 10^11^ Jones, and the rise and fall time were 29.76, 46.73 ms, respectively. Based on these results, the Ag_2_O/β-Ga_2_O_3_ heterojunction photodetector operates without an externally applied voltage and has high responsivity, which will help in the performance improvement of ultraviolet sensing systems.

## 1. Introduction

Ultraviolet (UV) radiation from the sun has been used in a variety of applications such as disinfection of air, surfaces, and instruments [1]. Depending on the wavelength, UV light can be divided into UV-A (320–400 nm), UV-B (280–320 nm), and UV-C (200–280 nm). Among them, UV-C light does not reach the ground level owing to strong ozone layer absorption at the earth’s surface. It is also known as solar-blind deep UV (DUV) radiation. In addition, DUV can catalyze chemical reactions, sterilize, and radiate from initial flame combustion. Owing to these characteristics, the need for DUV utilization has increased, and photodetector sensing DUV research has been extensively conducted in various fields, including biological and chemical analyses, solar UV monitoring, and flame sensors [2,3,4,5,6].

Proper materials for DUV photodetectors are ultra-wide bandgap semiconductors, such as ZnMgO and AlGaN [7,8]. However, the incorporation of significant amounts of Al in AlGaN deteriorates the crystal quality, and ZnMgO appears to be phase-separated [9,10]. Conversely, gallium oxide (β-Ga_2_O_3_), a well-known wide-bandgap semiconductor, has attracted considerable attention because it can fabricate high-quality thin films without other methods such as doping [11]. β-Ga_2_O_3_ has been extensively studied for DUV optoelectronics and power electronic devices because of its large direct bandgap energy (4.8 eV), high breakdown electric field (E_br_ = 8 × 10^6^ V/cm), and excellent chemical and thermal stability [12,13,14]. In addition, β-Ga_2_O_3_ exhibits n-type semiconductor properties owing to oxygen vacancies [15].

Most photodetectors require additional external power to promote the generation of photocurrents. This increases the size of the device, making it inconvenient to package and carry, and becomes overly dependent on external power supplies, limiting practical application in various negative complex environments. To complement these disadvantages, a new type of self-powered photodetector has been designed through extensive research. Small in size, economical, and featuring low energy consumption, these self-powered photodetectors do not depend on externally applied voltages and can be implemented independently of self-powered photodetectors. The response mechanism of this self-powered photodetector utilizes electron-hole pairs generated by light irradiation. i.e., It is based on the photovoltaic effect of semiconductors. The photodetector can be classified into a photoconductor and photodiode type according to the operating principle. However, the photodiode type is important because it implements a self-powered sensor with high responsivity depending on the material selection. According to photodiode construction mechanism, the current self-powered photodiode can be divided into two types: Schottky junction, and p-n heterojunction [16,17]. Schottky junctions have the advantages of fabrication simplicity and high-speed response. However, Schottky barriers built into the contacts of a Schottky barrier photodetector can make it difficult for the charge to transfer and degrades the device responsivity. The p-n heterojunction is important because of its low reverse saturation current, high breakdown voltage, and adjustment of the photodetection wavelength according to the heterojunction material [18]. Herein, suitable p-type semiconductors for the fabrication of photodetector devices as counterparts for n-type β-Ga_2_O_3_ are explored.

In this study, a photodetector based on a p-n junction was fabricated, and heterojunctions of n-type β-Ga_2_O_3_ and p-type Ag_2_O were investigated. Compounds of Ag with oxygen have been extensively investigated. Silver oxide has various phases, such as AgO, Ag_2_O, Ag_2_O_3_, Ag_4_O_3_, and Ag_4_O_4_ [19,20,21,22,23]. Among them, Ag_2_O is the most thermodynamically stable, and Ag_2_O thin films exhibit p-type semiconducting properties [24]. Various studies have been conducted on p-type Ag_2_O thin films owing to their excellent electrical properties and transparent properties in the infrared and visible ranges [25]. In addition, the transition from Ag to Ag_2_O by adjusting the oxygen flow ratio has previously been reported. Ag_2_O thin films can be fabricated by various techniques, such as thermal oxidation of Ag films [26], thermal evaporation [27], pulsed laser deposition [28], and sputtering [29,30]. In this study, an Ag_2_O layer was deposited using a facing target sputtering (FTS) system, which is a DC sputtering system. The FTS system consists of two targets facing each other within the chamber, and the substrate is separated from the plasma area, resulting in less surface damage compared to typical sputtering systems [31,32,33,34]. Based on the Ag_2_O/β-Ga_2_O_3_ heterojunction, we fabricated a self-powered solar-blind photodetector. It exhibited high performance in terms of photoresponsivity and DUV detectivity at zero bias, and it was confirmed that the Ag_2_O/β-Ga_2_O_3_ heterojunction photodetector operated with high performance without an external power supply.

## 2. Experimental Procedure

### 2.1. Materials

The solar-blind heterojunction photodetector was fabricated on an n-type single-crystal Sn-doped β-Ga_2_O_3_ wafer (thickness of 415 μm, N_d_-N_a_ = 4.50 × 10^18^ cm^−3^, Novel Crystal Technology, Inc.). Halide vapor phase epitaxy (HVPE) was used to grow the epitaxial layer of Si-doped β-Ga_2_O_3_ (thickness of 10 μm, N_d_-N_a_ = 2.20 × 10^16^ cm^−3^, μ_n_ = 300 cm^2^/Vs, ρ = 0.94 Ω cm), which was used as the active layer in this solar-blind heterojunction photodetector. This layer is used due to its high purity and provides low resistance and on-resistance, and a high breakdown voltage [35]. Ti/Au electrodes (10/40 nm) were deposited by sputtering on the backside of the Sn-doped β-Ga_2_O_3_ wafer for the ohmic device contact. For the deposition of the p-type Ag_2_O thin film, a 4–inch Ag targets (RND Korea, Gwangmyeong-si, Korea) and soda–lime glass substrate (75 × 25 cm, 1 mm thick, Marienfeld, Lauda-Königshofen, Germany) were used.

### 2.2. Fabrication of the Ag_2_O/β-Ga_2_O_3_ Heterojunction Photodetector

**Figure 1:**
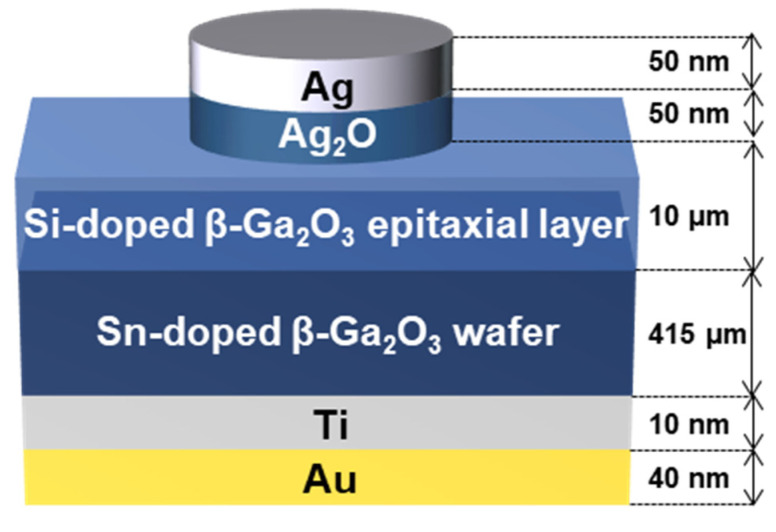
Schematic illustration of the fabricated Ag_2_O/β-Ga_2_O_3_ heterojunction photodetector.

Figure 1 shows a schematic illustration of the fabricated Ag_2_O/β-Ga_2_O_3_ heterojunction photodetector. Ag is used as the front photodetector electrode because it is capable of multilayer Ag_2_O deposition through continuous deposition. Ag_2_O/Ag (50/50 nm) thin films were patterned into circles with a 300 μm radius using a shadow mask on the top-epitaxial surface of the n-type β-Ga_2_O_3_ using an FTS system to fabricate an Ag_2_O/β-Ga_2_O_3_ heterojunction photodetector. The Ag_2_O thin film was deposited by controlling the oxygen flow, and the Ag thin film was continuously deposited without an oxygen atmosphere. The sputtering conditions are summarized in detail in Table 1. In addition, to improve the device performance of the fabricated photodetector, a post-annealing process was performed in the range from room temperature (RT) to 400 °C using rapid thermal annealing (RTA) in an Ar condition at 100 mTorr for 1 min.

The crystallographic properties of the thin films were evaluated using X-ray diffraction (XRD; Bruker D8) at the Smart Materials Research Center for IoT, Gachon University. Scanning electron microscopy (SEM, S-4700, Hitachi, Tokyo, Japan) at the Smart Materials Research Center for IoT, Gachon University and a KLA-Tencor Alpha-step D-500 Stylus Profiler were used to observe the surface morphologies and thicknesses of the thin films, respectively. The optical properties of the as-deposited thin films were evaluated using a UV-vis spectrometer (Lambda 750 UV-vis-NIR, Perkin Elmer, Waltham, MA, USA). The electrical properties of the thin films were evaluated by Hall effect measurement system (HMS-5500, ECOPIA, Anyang, Korea). X-ray photoelectron spectroscopy (XPS, K-alpha +, Thermo Fisher Scientific, Waltham, MA, USA) was used to investigate the binding energy of Ag_2_O before and after post-annealing.

### 2.3. Evaluation of the Ag_2_O/β-Ga_2_O_3_ Heterojunction Photodetector

The electrical current density-voltage (J-V) characteristics were analyzed using a semiconductor analyzer (4200A-SCS parameter analyzer, Keithley, Cleveland, OSU, USA). A Keithley 2401 was used to measure the time-dependent photoresponse of the fabricated device. Time-dependent photoresponse measurements were performed using a UV-C lamp (TN-4LC, wavelength: 254 nm, Korea Ace Scientific) with varying light intensities ranging from 100 to 1000 μW/cm^2^ at RT. The UV-C intensity was measured using a UV-C light meter (UVC-254SD, Lutron, PA, USA).

## 3. Results and Discussion

For the heterojunction photodetector fabrication, p-type Ag_2_O thin-film deposition was conducted. In reactive sputtering, a transition region representing the change from various metals to oxides through discharge voltage changes has been observed in numerous studies. For example, by changing the discharge voltage of Si, the metal target surface switches to the oxide mode when oxygen reacts with the metal particles and target surface. A transition region was observed, and the result of the conversion to SiO_2_ was reported in a previous study [36]. Therefore, to deposit p-type Ag_2_O, the discharge voltage change under different oxygen flows in the Ag target was measured.

**Figure 2:**
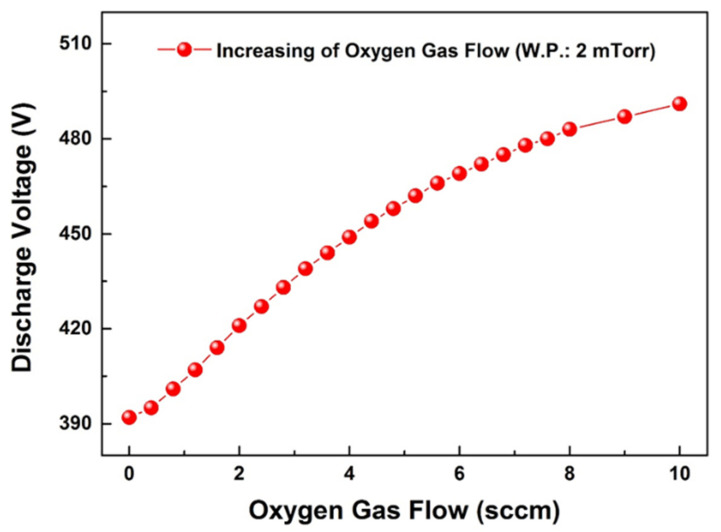
Variation of discharge voltage by changing the oxygen flow rate.

Figure 2 shows the variation in the discharge voltage with the oxygen flow rate in the Ag target. As the oxygen flow rate increased, the discharge voltage also increased; therefore, the transition region representing the change from metal to oxide was not observed. As a predictable cause of this trend, reactive gas such as oxygen can easily form negative ions in the plasma state. In the case of Si, because the emission coefficient of the secondary electrons emitted from the surface of the Si target is higher than that of oxygen gas, the discharge voltage decreases as the oxygen gas flow rate increases. However, in Ag, because the emission coefficient of secondary electrons emitted from the Ag metal target surface is lower than that of oxygen gas, the discharge voltage is expected to increase as the oxygen gas flow rate increases [37].

**Figure 3:**
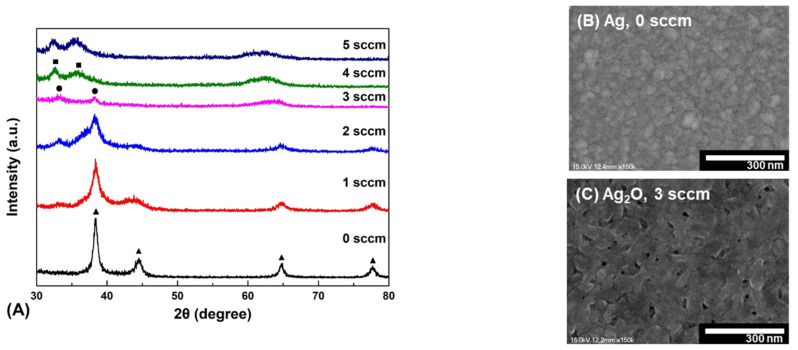
Structural properties of as-deposited thin film. (**A**) XRD patterns of as-deposited thin film under different oxygen gas flow rates. (●: Ag_2_O, ▲: Ag, ■: AgO). (**B**) SEM image of Ag thin film deposited on a glass substrate. (**C**) SEM image of Ag_2_O thin film.

Figure 3A shows the XRD patterns of as-deposited thin film by changing oxygen gas flow in the range of 0 to 5 sccm. Without the oxygen gas, the Ag peaks appeared at 2θ = 38.2°, 44.4°, 64.6°, and 77.6° corresponding to the (111), (200), (220), and (311) planes, respectively, according to the reference data (ICDD card 01-087-0720). At 2 sccm, the intensity of Ag peaks decreased and the (100) planes of Ag_2_O (ICDD card 01-072-2108) additionally appeared at 2θ = 33.6°, indicating a mixed phase of Ag_2_O and Ag. Moreover, at 3 sccm, the broad peaks representing the (100) and (011) planes of Ag_2_O appeared at 2θ = 33.6° and 38.4° without other impurities. The (011) peak of Ag_2_O and the (111) peak of Ag may overlap. However, it could be sufficiently distinguished through the additional peaks of Ag and Ag_2_O. As the oxygen gas flow increases from 4 to 5 sccm, the Ag_2_O peaks were not seen, and peaks corresponding to AgO (JCPDS card 75-0969) were observed at 2θ = 32.3° and 35.6°. It is suggested that with an increased oxygen gas flow rate, an additional chemical bond between Ag and O is generated, resulting in AgO peaks [38]. The AgO demonstrated deteriorated electrical properties compared to Ag_2_O, and its properties as a p-type are reduced [39]. Hence, our work was conducted at 3 sccm where only the Ag_2_O peak exists. In the top-view SEM images of Figure 3B,C, the surface morphologies of the Ag and Ag_2_O thin film indicate a dense surface.

**Figure 4:**
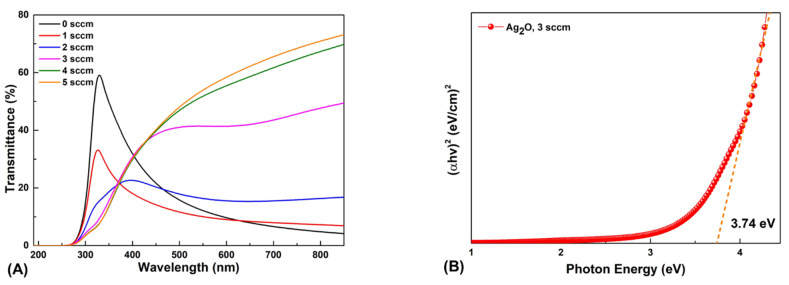
Optical properties of the as-deposited thin film prepared at different oxygen gas flow rates. (**A**) Transmittance (%) values of different samples at oxygen gas flow ranging from 0 to 5 sccm. (**B**) Optical bandgap energy of Ag_2_O thin film.

Figure 4A shows the UV-vis spectra of the as-deposited thin film with the oxygen gas flow rate controlled from 0 to 5 sccm. The transmittance near 300 nm of the Ag thin film deposited at 0 and 1 sccm increase sharply than other conditions. The reason is the surface plasmon resonance (SPR) phenomenon that occurs on the surface of planar metals (typically gold or silver) or on the surface of metal nanoparticles. Therefore, it is a SPR phenomenon that appears on the surface of the Ag thin film through the light used for UV-vis, which increases the transmittance near 300 nm [40]. As the oxygen flow rate increased, the transition from Ag to Ag_2_O occurred, increasing the transmittance in visible light of the thin film more than the Ag thin film [41]. Moreover, the average transmittance in visible light of the thin film deposited at 3 sccm was significantly increased to 42.5%. Additionally, when the oxygen gas flow was increased to 4–5 sccm, it was confirmed that the average visible light transmittance increased. However, as mentioned in the XRD study, the AgO phase appeared at the 4–5 sccm oxygen gas flow condition. Therefore, the optimal condition was chosen as 3 sccm.

Figure 4B shows the optical bandgap energy of the Ag_2_O thin film calculated by the Tauc plot equation [42], given by

*αhv* = *β*(*hv* − *E_g_*)^1/2^ (1)

where *hν* is the photon energy, *α* is the absorption coefficient, and *β* is a fixed constant. The Tauc plot shows that the optical bandgap energy can be estimated by extrapolating the linear section to the axis energy. The optical bandgap energy of the Ag_2_O thin film deposited at 3 sccm was calculated to be 3.74 eV. Electrical properties of as-deposited Ag_2_O thin film were evaluated using Hall measurement. The carrier concentration, hall mobility, and resistivity of as-deposited Ag_2_O thin film are 6.2 × 10^18^ cm^−3^, 41.5 cm^2^/Vs, and 2.4 × 10^−2^ Ω cm, respectively. Moreover, the positive value of Hall coefficient of 1.0 cm^3^/C was obtained, confirming that the as-deposited Ag_2_O thin film was a p-type semiconductor.

The influence of post-annealing on the Ag_2_O properties was also evaluated. It has been reported that an appropriate post-annealing temperature improves the photodetector characteristics owing to a reduction in interfacial defects [43]. The crystallinity quality, uniformity of the deposited thin film, and native defect correction at the heterojunction interface resulted in an increased photocurrent or apparent photoresponse for the fabricated device. Because the deposited Ag_2_O thin films are affected by electrical and crystallographic properties depending on the post annealing temperature, the as-deposited Ag_2_O thin films were studied in the temperature range RT−600 °C to evaluate the variation in Ag_2_O crystallographic properties.

**Figure 5:**
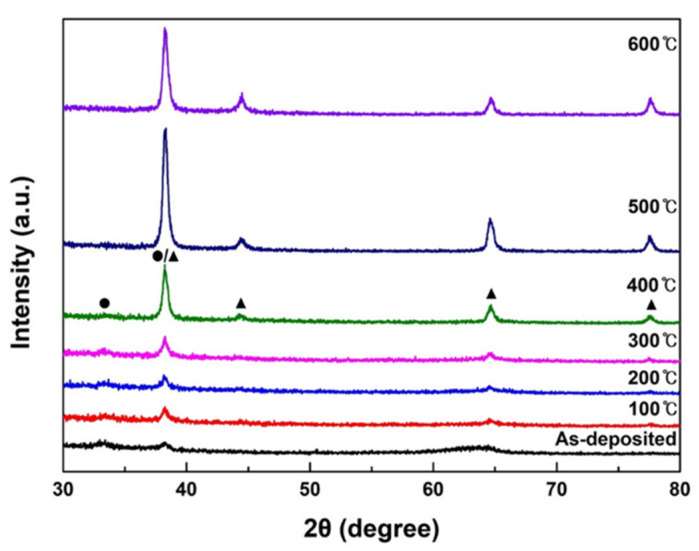
XRD patterns of the post-annealed Ag_2_O thin films in the range of RT to 600 °C. (●: Ag_2_O, ▲: Ag).

The XRD patterns of the post-annealed Ag_2_O thin films are shown in Figure 5. The annealing temperature range was from RT to 600 °C. Broad peaks appeared in the Ag_2_O thin film deposited at RT, and as the post-annealing temperature increased, the Ag_2_O peak intensity increased. The intensity of (011) peak at 38.4°, the preferred growth plane, increased the most at 300 °C, without crystal transition from Ag_2_O to Ag. The Scherrer equation was used to calculate the crystallite sizes of the Ag_2_O (011) plane according to the annealing temperature [44]. It was confirmed that the crystallite size increase with annealing temperature (pristine: 13.3 nm, 100 °C: 14 nm, 200 °C: 15 nm, 300 °C: 16.5 nm). As the heat treatment temperature increases, it becomes a high-quality thin film with improved crystallinity, and the surface of the film becomes homogenous due to reduced roughness. The smooth surface exhibits good interfacial properties and can effectively suppress the formation of interfacial charge traps and reduce the carrier scattering centers to achieve greater electron mobility, which greatly improves the performance of the photodetector [45]. However, after heat treatment at 400 °C, the intensity of Ag_2_O (100) peak was slightly decreased, and it was confirmed that the intensity of the Ag peaks (200), (220), and (311) increased. This means that the sample post-annealed at 400 °C presents both Ag_2_O and Ag phases. These thin films have inhomogeneous surface which cause more traps and dislocations at the Ag_2_O/Ag and Ag_2_O/β-Ga_2_O_3_ interfaces. Since these defects acts as a resistance in the diode characteristics, it is expected that such as hump phenomenon will appear. The transition of Ag_2_O into Ag is attributed to the increase in the Ag diffusion rate owing to the thermal effect, and the diffusion rate of oxygen atoms becomes relatively smaller than that of Ag. Therefore, owing to sufficient thermal energy, the Ag diffusion increases and the chemical bond with oxygen is broken [46]. Thus, at 500 °C or higher, the Ag_2_O peaks were not observed and the Ag peaks appeared. Moreover, the core level binding energy was further investigated through XPS analysis of the post-annealed sample at 400 °C, in which both Ag_2_O and Ag peaks exist.

**Figure 6:**
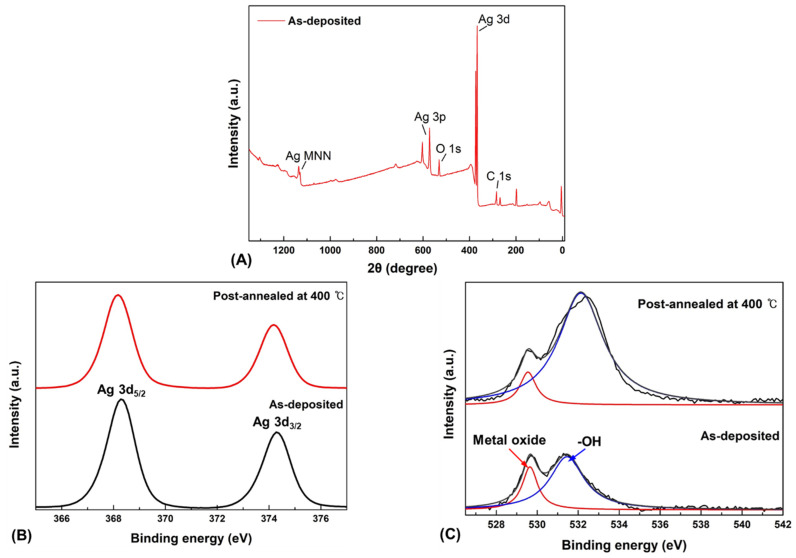
XPS spectra of the Ag_2_O thin films with and without post-annealing. (**A**) Full XPS spectrum of Ag_2_O thin film, (**B**) Ag 3d, (**C**) O 1s.

Figure 6 shows the XPS spectra of Ag_2_O thin films with and without post-annealing. The XPS analysis was performed to further investigate the changes in Ag_2_O film properties post-annealed at 400 °C via the core-level binding energy. The chemical composition of Ag_2_O was determined by XPS. As presented in Figure 6A, the full XPS spectrum of Ag_2_O reveals the presence of C, O, and Ag. Through the XPS analysis, the atom ratio between Ag and O was 2:1. However, after heat treatment at 400 °C, the atom ratio of Ag and O is changed to 3.5:1. Since the atom ratio of Ag becomes higher than that of O, it can be seen that Ag_2_O is transitioning to Ag. The spectrum in Figure 6B shows the characteristic core-level binding energies at approximately 368 and 374 eV, corresponding to Ag 3d_5/2_ and Ag 3d_3/2_, respectively, related to the Ag_2_O thin films. The core-level binding energies shown in the XPS spectra are consistent with those reported in other studies [47]. Moreover, after post-annealing at 400 °C, the core level binding energy of Ag 3d_5/2_ was shifted to 368.3 eV and Ag 3d_3/2_ shifted to 374.3 eV. In the O 1s XPS spectrum in Figure 6C, the two peaks at 529.5 and 531.4 eV are related to the core-level binding energy of the O ion in Ag_2_O, whereas the higher-energy peak corresponds to hydroxyl groups (-OH). Compared with thin film post-annealed at 400 °C, the core-level binding energy of the O ion shifted to 529.4 eV and -OH shifted to 532.1 eV. This indicates that the transition from Ag_2_O to Ag was caused by thermal decomposition [48]. As shown in Figure 6, the sample without post-annealing indicated Ag_2_O. However, the post-annealing of the 400 °C sample showed an Ag_2_O to Ag transition region. These XPS results are in good agreement with the XRD results. Hence, heat treatment was performed on the photodetector in the range of 0–400 °C, where the Ag_2_O peaks were maintained.

**Figure 7:**
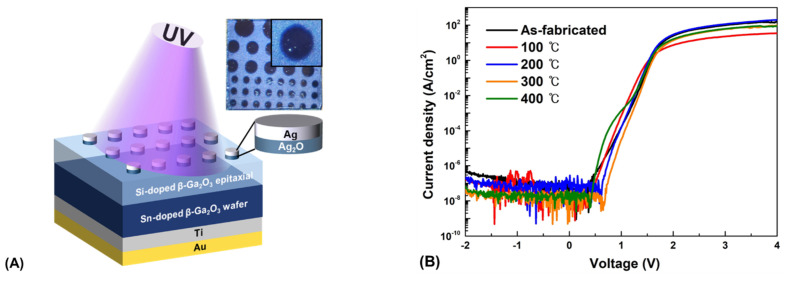

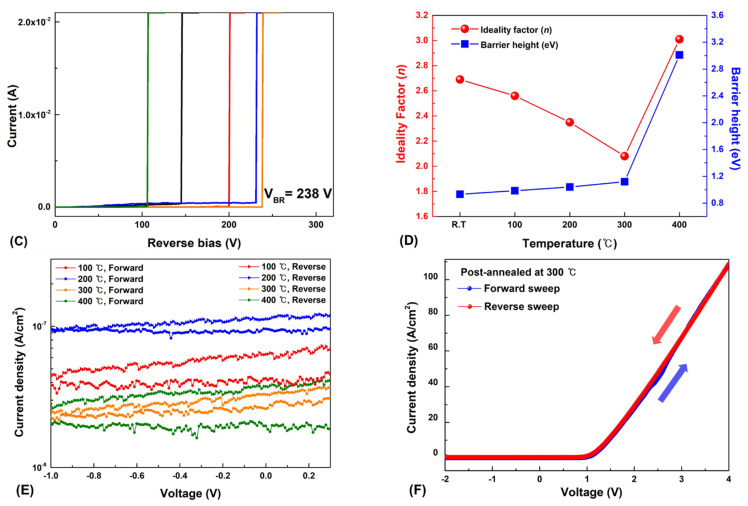
(**A**) Schematic illustration of the as-fabricated Ag_2_O/β-Ga_2_O_3_ heterojunction photodetector. (**B**) J-V characteristics of Ag_2_O/β-Ga_2_O_3_ heterojunction photodetectors. (**C**) Breakdown voltage of the as-fabricated devices. (**D**) Variation of the ideality factor (*n*) and barrier height (*φ_B_*) with the post-annealing temperature. (**E**) Logarithmic plot of the J-V characteristics with difference in reverse saturation current according to post-annealing temperature. (**F**) J-V characteristics for the forward and reverse sweep of bias voltage.

After optimizing the Ag_2_O condition, an Ag_2_O/β-Ga_2_O_3_ heterojunction photodetector was fabricated, and its structure is shown in Figure 7A. The J-V characteristics of the Ag_2_O/β-Ga_2_O_3_ heterojunction photodetectors fabricated at various post-annealing temperatures were measured in the range of −2–4 V are shown in Figure 7B. All devices exhibited p-n junction rectification characteristics; in particular, a high on-off ratio of 1.40 × 10^8^ was achieved through a low off-current of 4.24 × 10^−11^ A at 0 V and an on-current of 5.74 × 10^−3^ A at 1.86 V in the device post-annealed at 300 °C. Moreover, the J-V curve of the fabricated device without heat treatment indicates that the leakage current increases as the reverse bias voltage increases. However, the leakage current decreased as the post-annealing temperature increased. The change in leakage current with annealing temperature may influence the Ag_2_O physical properties at different annealing temperatures. Post-annealing of the layers can improve the interface properties between the heterojunctions; thus, the homogeneity of the junction reduces carrier recombination and leakage current [49]. As shown in Figure 7C, it exhibited the variation of the breakdown voltage with post-annealing temperature. The breakdown voltage increased as the annealing temperature increased, and the device post-annealed at 300 °C exhibited the highest value at 238 V. The photodetector is primarily used at low current, but the as-fabricated device has high durability owing to its high breakdown voltage and low leakage current, such that it can be applied in harsh industries where a sudden short circuit occurs [50]. In addition, diode parameters such as the ideality factor and barrier height were investigated. The diode J-V characteristics can be expressed, correlating the current and voltage with the ideality factor and barrier height, as follows:

*J* = *J_S_* exp{*q*(*V* − *IR_S_*)**/***nkT*} (2)

where *J_S_* is the saturation current density, *V* is the voltage across the diode, *T* is the temperature in Kelvin, *q* is the electron charge, *k* is Boltzmann’s constant, and *n* is an ideality factor. The value of the ideality factor determines the deviation from the ideal diode owing to the presence of the barrier inhomogeneity and tunneling component [51],

*J_S_* = AA*T^2^exp(−*qφ_B_*/*kT*) (3)

where A* is Richardson’s constant (41 A cm^−2^ K^−2^ for β-Ga_2_O_3_), *A* is the contact area, *T* is the temperature in Kelvin, *q* is the electron charge, *k* is Boltzmann’s constant, and *φ_B_* is the effective barrier height.

Figure 7D shows the changes in *n* and *φ_B_* with the post-annealing temperature. As the post-annealing temperature increased up to 300 °C, the value of *n* decreased and *φ_B_* increased. The ideality factor of the device post-annealed at 300 °C had the lowest value of 2.08, and *φ_B_* increased to 1.12 eV. This can be attributed to improvements in crystallization and reduced interfacial defects with fewer dislocations because of post-annealing [52]. However, the values of *n* and *φ_B_* increased rapidly at 400 °C, this is due to the hump phenomenon observed in the J-V curve in Figure 7B. This was related to the transition of Ag_2_O to Ag caused by the post-annealing process at 400 °C. As shown in the XRD study, the thin film post-annealed at 400 °C indicates a mixed state of Ag_2_O and Ag. It is thought that the trap density increases owing to the trapping of charged species occurring from the difference in the resistivity and conductivity of the mixed crystallinity, causing the hump phenomenon [53]. Furthermore, the interface quality of the Ag_2_O/β-Ga_2_O_3_ heterojunction was investigated. The occurrence of electrical hysteresis was analyzed by measuring the forward and reverse J-V characteristics. Figure 7E shows a logarithmic plot of the J–V characteristics with small electrical hysteresis at the reverse saturation current according to the post-annealing temperature. The presence of electrical hysteresis in the reverse saturation current exhibits the quality of interface states and surface defects for the Ag_2_O/β-Ga_2_O_3_ heterojunction [54,55]. It was confirmed that the difference in reverse current and the reverse saturation current decreased as the post-annealing temperature increased, showing the narrowest gap at 300 °C. As the difference in reverse current and reverse saturation current is much smaller, charge trapping is reduced, indicating an enhanced interface state. Figure 7F shows the forward and reverse sweeps of the post-annealed device at 300 °C under dark condition. The measured J–V characteristics indicated almost no hysteresis effect compared to the forward and reverse, showing an excellent interface state.

**Figure 8:**
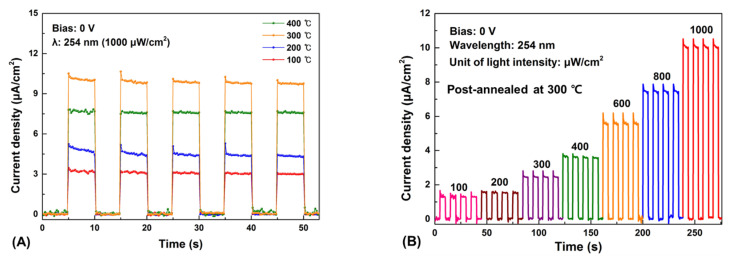

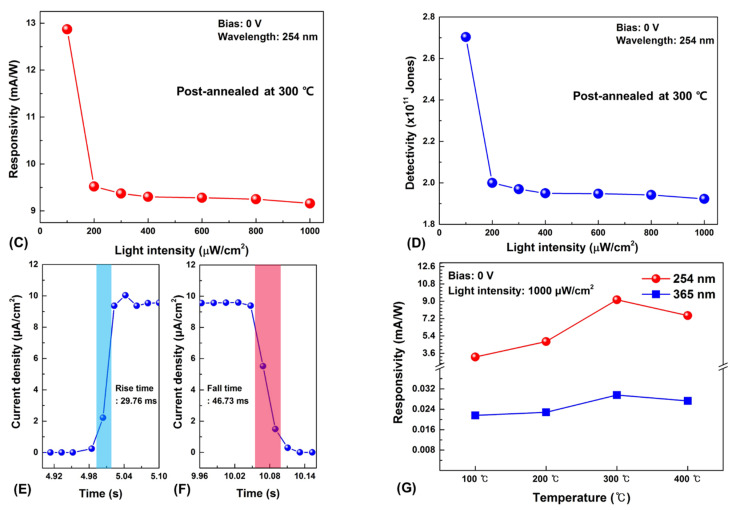
The photoresponse and parameters of the Ag_2_O/β-Ga_2_O_3_ heterojunction photodetector under zero-bias at 254 nm irradiation. (**A**) Photoresponse of the devices with the Ag_2_O thin films grown under different post-annealing temperatures. (**B**) Photoresponse of the device post-annealed at 300 °C with various light intensities. (**C**) Responsivity as a function of the light intensity. (**D**) Detectivity as a function of the light intensity. (**E**) Rise time and (**F**) Fall time. (**G**) Responsivity under zero-bias at 254 and 365 nm irradiation.

Figure 8A shows the continuous time-dependent photoresponse according to the post-annealing temperature at a wavelength of 254 nm and a light intensity of 1000 μW/cm^2^ at zero bias voltage. When the UV light was turned on, the photocurrent instantaneously flowed, and the photoresponse of all the samples remained approximately the same, indicating high reproducibility. Moreover, when the UV light was turned off, the photocurrent immediately returned to the dark current. This on/off operating characteristic of the UV irradiation clearly indicates that it behaves as a photodetector. Moreover, the effect on heat treatment was examined, the photocurrent increased with increasing post-annealing temperature, showing the highest photocurrent density of 9.70 μA/cm^2^ at 300 °C. In the case of 400 °C, the photocurrent decreased owing to the hump phenomenon, in which Ag_2_O transitions to Ag caused by post-annealing at 400 °C. The change in photocurrent density according to post-annealing temperature tends to be the same as the J-V analysis. The interfacial traps interfere the movement of electrons separated from a pair of electron holes generated by light irradiation. However, it can be seen that the interfacial traps reduced at 300 °C through the J-V analysis and photoresponse according to the heat treatment, thereby stabilizing the interface between Ag_2_O and β-Ga_2_O_3_ and improving the quality. In addition, temperature-dependent external quantum efficiency (EQE) was calculated using the following formulas:

*η*_eff_ = (*hvR*/*e*) × 100% (4)

where *η*_eff_ is the EQE, *hv* is the incident photon energy, *R* is the responsivity, and *e* is elementary charge.

Comparing the values of *η*_eff_ for each annealing temperature, it was confirmed that the device post-annealed at 300 °C achieved the highest *η*_eff_ of 6.3% (100 °C: 1.9%, 200 °C: 2.8%, 300 °C: 6.3%, 400 °C: 4.8%) at zero-bias under 254 nm irradiation at 100 μW/cm^2^. Hence, it was confirmed that 300 °C is the optimal condition, the change in photocurrent of the 300 °C sample according to the intensity of light at 254 nm was investigated. Figure 8B shows the photoresponse of the post-annealed device at 300 °C under a UV light intensities ranging from 100 to 1000 μW/cm^2^ under 254 nm irradiation. The photocurrent gradually increased with the intensity of light because the higher the light intensity, the more electron-hole pairs, which in turn produced a higher photocurrent. However, contrary to this tendency, the responsivity and detectivity of the photodetector exhibited different results. Figure 8C,D shows the change in the photodetector responsivity and detectivity according to the light intensity. The responsivity, which evaluates the photodetector sensitivity and the detectivity, which is the figure of merit for detecting the smallest signal, were calculated using the following formulas [56,57]:

*R* = (*J_Photo_* − *J_Dark_*)/*P* (5)

where *R* is the responsivity, *J_Photo_* is the photocurrent density, *J_Dark_* is the dark-current density, and *P* is the supplied light power. The detectivity value is determined by,

*D* = *R*/(2*eJ*)^1/2^ (6)

where *D* is the detectivity, *J* is the dark-current density, and *e* is the elemental charge.

Consequently, the responsivity under 254 nm irradiation decreased with increasing light intensity. The maximum responsivity was obtained as 12.87 mA/W at 100 μW/cm^2^. Moreover, the detectivity decreased with increasing light intensity, and the highest *D* was obtained at 2.70 × 10^11^ Jones under 254 nm irradiation at 100 μW/cm^2^. The device exhibited high responsivity and detectivity at low light intensities. For this reason, it is suggested that as more electron-hole pairs are generated by high light intensity, scattering is increased because of the generated charge carriers, and the possibility of recombination increases, thereby reducing responsivity and detectivity [58]. Figure 8E,F show the rise and fall times of the Ag_2_O/β-Ga_2_O_3_ heterojunction photodetector. It was measured to be 29.8 and 46.7 ms, respectively. As shown in Figure 8G, we confirmed that the rejection ratio of 254 nm (UVC) to 365 nm (UVA) at zero bias under the light intensity of 1000 μW/cm^2^ is 3.1 × 10^2^. The photoresponse parameters under zero bias of the Ag_2_O/β-Ga_2_O_3_ heterojunction photodetector in this study showed higher responsivity and detectivity than those of other solar-blind photodetectors, as shown in Table 2 [59,60,61,62,63,64,65].

**Figure 9:**
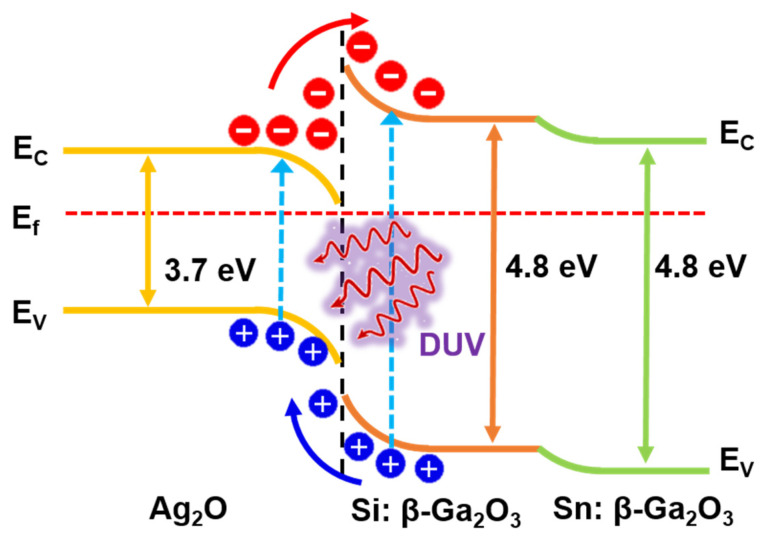
Energy band diagram of the Ag_2_O/β-Ga_2_O_3_ heterojunction under DUV light irradiation.

The photoresponse properties of the Ag_2_O/β-Ga_2_O_3_ photodetector can be explained from the energy band diagram as shown in Figure 9. The mechanism of photocurrent generation is related to the depletion region. For p-type Ag_2_O, in which positive holes are the major carriers, the Fermi level is located near the valance band, whereas n-type β-Ga_2_O_3_, in which free electrons are the major carriers, exists near the conduction band. When forming the p-n junction holes from p-type diffuse to n-type, and electrons from n-type diffuse to p-type. As a result, each Fermi level is matched, forming an electrically neutral depletion region. In addition, due to the large difference in optical bandgap, a strong built-in electric field will be created at the Ag_2_O/β-Ga_2_O_3_ interface. When the device is irradiated to DUV light, electron–hole pairs are generated in the depletion region due to absorption of the incident DUV light, which is rapidly separated by the strong built-in electric field. Moreover, electrons are transferred to the electrode, leading to a fast photoresponse speed and an increase in photocurrent. Besides, the strong built-in electric field ensures that this heterojunction photodetector can operate at zero bias and proper heat treatment reduces defects between interfaces, resulting in low leakage currents and high photoresponse. In this study, we successfully fabricated a self-powered DUV photodetector through the heterojunction of Ag_2_O and β-Ga_2_O_3_. The photodetector characteristics according to the post-annealing temperature were evaluated, and the sample that was post-annealed at 300 °C had the best performance. Our research provides a new route for the DUV sensing industry and shows potential for military applications, including flame sensing, spatial sensing, and image sensors.

## 4. Conclusions

A self-powered solar-blind photodetector was successfully fabricated with an Ag_2_O/β-Ga_2_O_3_ heterojunction via deposition of a p-type Ag_2_O thin film onto an n-type β-Ga_2_O_3_ layer using an FTS system. Ag_2_O thin films were deposited by controlling the oxygen gas flow, with 3 sccm being the optimal condition for presenting p-type semiconductor characteristics. After the Ag/Ag_2_O thin film deposition, the effects of post-annealing on the Ag_2_O/β-Ga_2_O_3_ heterojunction photodetector were investigated. Typical rectification characteristics were observed in J-V characteristics, and as the post-annealing temperature increased to 300 °C, a low leakage current (4.24 × 10^−11^ A), high breakdown voltage of 238 V, ideality factor of 2.08, and barrier height of 1.12 eV were observed. A high responsivity of 12.87 mA/W, detectivity rate of 2.70 × 10^11^ Jones, and fast rise and fall times of 29.76 and 46.73 ms were observed under 254 nm irradiation at zero bias. From these results, it was confirmed that the device operates without an external applied voltage and has high responsivity in DUV. Ag_2_O/β-Ga_2_O_3_ heterojunction photodetectors have confirmed their potential for application in DUV sensing systems, and further research is planned to improve the device characteristics.

## Figures and Tables

**Table 1:** Sputtering conditions of Ag_2_O and Ag thin films.

Parameters	Sputtering Condition	
Layer	Ag_2_O	Ag
Substrate	Soda-lime glass	Soda-lime glass
Targets	Ag (99.99%)	Ag (99.99%)
Base pressure	3 × 10^−5^ Torr	3 × 10^−5^ Torr
Working pressure	2 mTorr	2 mTorr
Gas flow	Ar: 10 sccm, O_2_: 3 sccm	Ar: 10 sccm
Input power	50 W	50 W
Thickness	50 nm	50 nm

**Table 2:** Comparison of the self-powered solar-blind photodetector characteristics parameters with other reported studies.

Photodetector	Wavelength (nm)	Responsivity (mA/W)	Detectivity (Jones)	Rise Time/Fall Time	Ref.
Ag_2_O/β-Ga_2_O_3_	254	12.87	2.70 × 10^11^	29.76 ms/46.73 ms	This work
Diamond/β-Ga_2_O_3_	244	0.2			[59]
Au/β-Ga_2_O_3_	258	0.01		1 μs/100 μs	[60]
4H-SiC/β-Ga_2_O_3_	254	10.35	8.8 × 10^9^	11 ms/19 ms	[61]
Polyanline/MgZnO	250	0.16	1.5 × 10^11^	0.3 s/0.3 s	[62]
p-Gr/ZnS QDs/4H-SiC	250	0.29	1.41 × 10^10^	28 μs/0.75 ms	[63]
MoS_2_/β-Ga_2_O_3_	245	2.05	1.21 × 10^11^		[64]
β-Ga_2_O_3_/Ga:ZnO	254	0.76		0.18 s/0.27 s	[65]

## Data Availability

The data that support the findings of this study are available from the corresponding author upon reasonable request.
